# Are Follicular Regulatory T Cells Involved in Autoimmune Diseases?

**DOI:** 10.3389/fimmu.2017.01790

**Published:** 2017-12-11

**Authors:** Yonglu Gong, Jia Tong, Shengjun Wang

**Affiliations:** ^1^Department of Laboratory Medicine, The Affiliated People’s Hospital, Jiangsu University, Zhenjiang, China; ^2^Institute of Laboratory Medicine, Jiangsu Key Laboratory of Laboratory Medicine, Jiangsu University, Zhenjiang, China

**Keywords:** follicular regulatory T cell, follicular helper T cell, B cell, germinal center, autoimmune disease

## Abstract

In the germinal center (GC), follicular helper T (TFH) cells interact with B cells and undergo a series of GC reactions to ultimately produce high-affinity antibodies and memory plasma cells. Recent studies have found a subpopulation of regulatory T cells called follicular regulatory T (TFR) cells. TFR cells can inhibit TFH cells and/or B cells in a variety of ways to specifically regulate GC reactions. Dysfunction of TFR cells may lead to immune disorders and a variety of autoimmune diseases. In this review, we summarize the differentiation and function of TFR cells and provide an overview of TFR cells in autoimmune diseases.

## Introduction

Follicular helper T (TFH) cells are specific CD4+ T cells that are generated from naive CD4+ T cells in the periphery of the T cell region of a secondary lymphoid organ and mediate antigen-specific naive or memory B cell activation in the follicles of the secondary lymphoid organ (1, 2). The interaction of TFH cells with activated B cells plays a key role in the processes of germinal center (GC) reactions (3, 4). In the GC, TFH cells provide survival and differentiation signals to B cells *via* the binding of CD40–CD40L and the secretion of interleukin (IL)-4 and IL-21. Enhanced B cell receptor signaling may also contribute to this reaction in the GC, which ultimately contributes to B cell differentiation into plasma cells (5–7). TFH cells play a key role in B cell activation and antibody production, and their inability to maintain immune homeostasis may lead to immune-mediated disease. GC reactions must be regulated to prevent the production of autoantibodies, systemic autoimmune diseases, chronic inflammation, allergic reactions, and the development of B cell malignancy (8–12).

In 2004, follicular regulatory T (TFR) cells were first discovered in human tonsils. A TFR cell is described as a specific type of regulatory T (Treg) cell capable of expressing CXCR5, Bcl-6, PD-1, and ICOS; thus, its phenotype is similar to that of TFH cells (13). An increasing number of studies have found that TFR cells can enter the B cell follicle and then specifically suppress TFH cells and B cells to control the GC reaction (14–16). TFR cell-mediated modulation of TFH and B cell interactions is necessary for a proper GC reaction, and abnormalities in the number or function of TFR cells can result in disorder of the GC reaction, which may lead to the development of an autoimmune response.

## Differentiation and Development of TFR Cells

**Figure 1:**
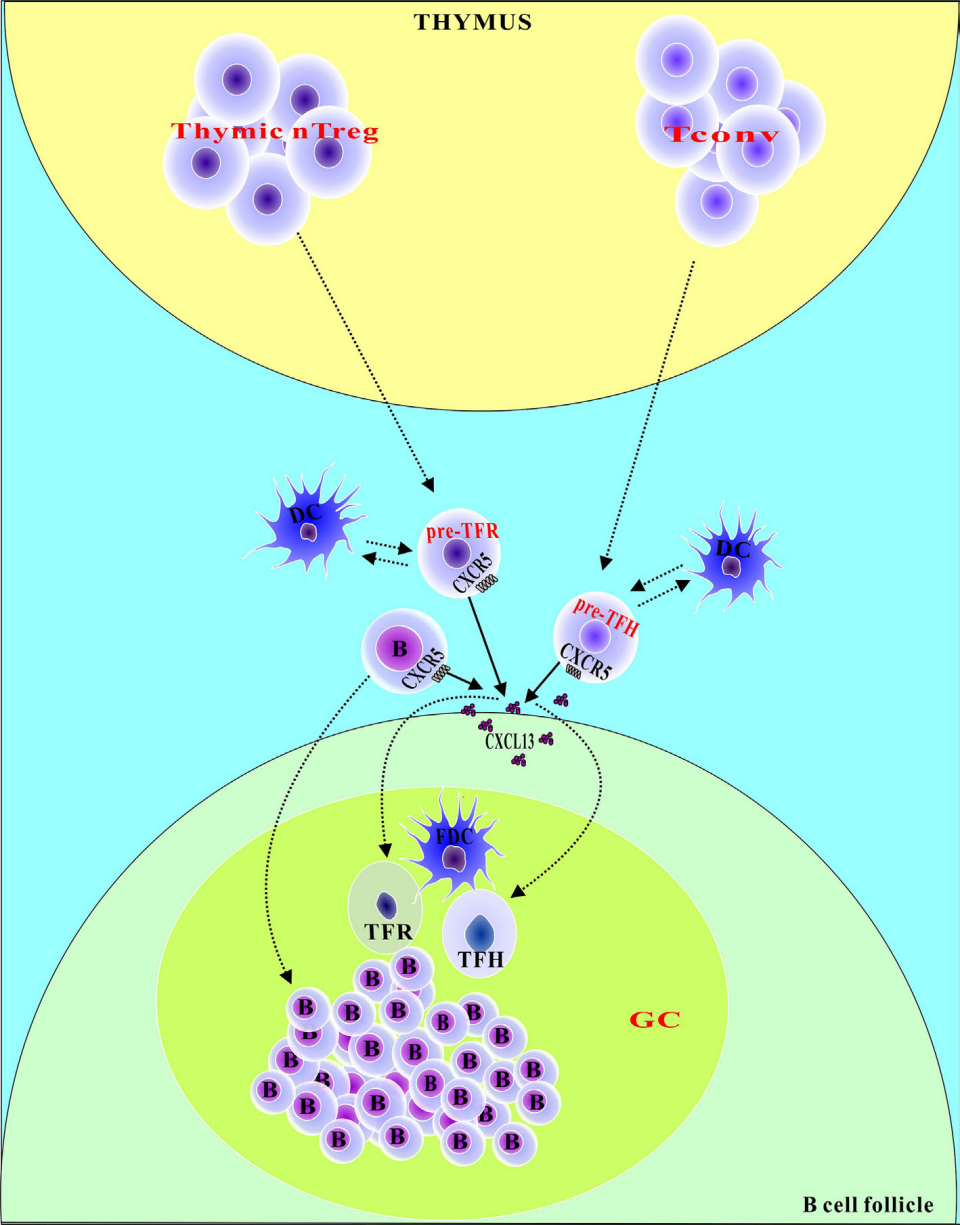
TFR and TFH cell ontogeny. TFH cells originate from conventional CD4+ T (Tconv) cells, whereas TFR cells originate mainly from thymic nTreg cells. TFH and TFR cells exit the thymus into the lymph fluid by bypassing the B-cell zone and have lower expression of ICOS and PD-1 than do germinal center (GC) TFH and TFR cells, which are called pre-TFH and pre-TFR cells, respectively. The CXCR5 molecule is expressed by pre-TFR and pre-TFH cells and senses gradients of CXCL13, directing pre-TFR and pre-TFH cells into the GC. The full differentiation of TFR or TFH cells occurs only after their interaction with cognate B cells and follicular dendritic cell cells within the follicle/GC.

TFR cells are derived from Treg precursor cells (Figure 1). Nevertheless, there is some debate over whether TFR cells are generated in the thymus or in peripheral lymphoid organs. In an *in vivo* study, Linterman et al. found that thymic Treg (nTreg) cells were capable of turning into TFR cells and that more than 97% of cells observed to do so expressed Helios (16). However, Chung et al. found that TFR cells were absent in the thymus but could be generated from CXCR5−Foxp3+ natural Treg precursors in the periphery (17). Moreover, Fonseca et al. found that CXCR5-expressing Treg cells were absent in human thymus and neonatal cord blood, suggesting that additional activation signals that are required to shape a CXCR5 phenotype in circulating Treg cells are not present before birth (18). It may be that Treg precursor cells that are generated in the thymus cannot become TFR cells in the thymus. In this scenario, these Treg precursor cells, which have retained some molecules formed in the thymus, such as CD31 and Helios, might migrate to peripheral lymphoid organs that possess a special microenvironment that is necessary for the development of TFR cells and there begin to differentiate into mature TFR cells. Treg precursor cells from lymphoid organs, such as the lymph nodes, Peyer’s patches, and spleen, differentiate into TFR cells in response to a variety of stimuli. These stimuli include the following: sheep red blood cells (SRBCs), foreign antigens such as OVA or keyhole limpet hemocyanin in adjuvant, self-antigens such as myelin oligodendrocyte glycoprotein (MOG), and viruses including lymphocytic choriomeningitis virus (LCMV) and influenza virus (13, 16, 17). FOXP− T precursor cells can also differentiate into TFR cells *via* PD-1L pathways in certain conditions (e.g., incomplete Freund’s adjuvant) (19). Similar to TFH cells, TFR cells require the help of dendritic cells (DCs) and B cells during development (8, 20, 21). It has been reported that TFR cells in the draining lymph nodes (dLN) and blood of mice with knocked out DCs are significantly reduced after immunization. After immunization of a μMT mouse that lacked B cells, TFR cells were found to be reduced in dLNs. However, there was no difference in TFR cells in the blood. The development of TFR cells in dLNs or blood is also different, indicating the need for B cells (20). Furthermore, in a study of patients receiving rituximab treatment (an anti-CD20 monoclonal antibody that knocks out B cells), the maintenance of TFH cells and TFR cells was found to not necessarily depend on B cells (15). TFR cells in human peripheral blood are generated in peripheral lymphoid organs; they do not interact with T-B, and they are not fully competent TFR cells. TFR cells of human peripheral blood maintain the ability to suppress T cell proliferation; however, they lack full B cell-suppressive capacity (18). The differences in TFR cells between humans and mice are unclear, and further exploration is needed. TFR cells and TFH cells have many similarities in the expression of relevant protein molecules such as PD-1, BCL-6, CXCR5, and ICOS that play very important roles in the development and function of TFR cells (14, 16, 17). It has been reported that the lymph nodes and peripheral blood TFR cells in PD-1 knockout mice increase after immunostimulation and that PD-1-deficient TFR cells have the potential to inhibit T cell activation. These findings indicate that the differentiation and function of TFR cells are affected by PD-1 (22, 23). CXCR5 (follicular guide) is a receptor for chemokine follicular CXCL13, gradients of which direct TFH cells and TFR cells to migrate to GC (24–27). In addition, BCL-6 is essential for the expression of CXCR5 on the surface of TFH cells. Similarly, the expression of BCL-6 on TFR cells is important for the expression of CXCR5 in TFR cells. It has been reported that BCL-6 can regulate the expression of CXCR5 on the surface of TFR cells to allow TFR cells to enter the follicle to regulate the GC reaction (17, 28).

Many factors regulate the expression of CXCR5 on the surface of TFR cells. It has been reported that NFAT-2 is expressed in a large number of follicular T cells (29). When NFAT-2 is knocked out in mouse CD4+ T cells, the GC reaction is enhanced, and the TFR cells in the GC are reduced. Previous work found that the expression of CXCR5 on the surface of FOXP3+ nTreg cells was upregulated and specifically dependent on NFAT-2, indicating that NFAT-2 is involved in the expression of CXCR5 on the TFR cell surface (29). BCL-6 also promotes the differentiation and development of TFR cells, which is similar to its effects on TFH cells (17). Blimp-1, as an inhibitor of BCL-6, plays a role opposite that of BCL-6 in the differentiation of TFR cells. After knocking out the *Prdm1* gene encoding the Blimp-1 protein in mice, TFR cells in the spleens of mice were significantly increased compared with their numbers in the spleens of wild-type (WT) mice, showing that the Blimp-1 protein plays a role in suppressing the differentiation of TFR cells (16). Moreover, Yang et al. found that Bilimp-1 was involved in mediating the suppression of TFR cells (30).

Although TFR and TFH cells have many phenotypic similarities, differences exist between them. Maceiras et al. found that the TFH and TFR pools in the GC were generated from distinct T cell receptor (TCR) repertoires. The TCR expressed on the surface of TFR cells is closer to that expressed on the surface of Treg cells, which leads to functional differences. The expression of TCR on TFH cells tends to promote antibody responses, whereas that on TFR cells tends to inhibit autoimmunity and has a function similar to Treg cells (31).

Similar to Treg cells, TFR cells express FOXP3, CTLA-4, GITR, and other molecules. It was reported that TFR cells in mice significantly increased after knocking out CTLA-4 molecules on the surface of mouse Treg cells but that most of their inhibitory function was lost (32). TFR cells suppress the expression of CD80 and CD86 on the surface of B cells *via* CTLA-4 molecules, which play a role in regulating the GC reaction (32). STAT3 is equally important for TFR cell differentiation. Knockout of the STAT3 gene in mouse T cells led to mouse TFR cell differentiation disorder and promoted IgG1 and IgG2b production by a large number of GC B cells. However, it did not have an effect on the number of TFH cells or B cells, which might be due to the involvement of STAT3 in the differentiation of TFR cells. Furthermore, STAT3 is involved in a variety of signaling pathways that affect the influencing factors of TFR cells (33). Sage et al. found that numbers of TFR cells were greatly decreased in the blood and lymph nodes of CD28−/− and ICOS−/− mice. It is likely that CD28 and ICOS provide essential costimulatory signals for the development of TFR cells in blood as well as in the lymph nodes (22). Building on the work by Sage et al., Chang et al. found that ICOS expression in Treg cells is essential for the formation and function of TFR cells. In addition, they found that after knockout of the gene encoding the TRAF3 protein in mice, the expression of ICOS in Treg cells was downregulated and the frequency of TFR cells was reduced after immunization (34). It was verified that TRAF3 plays a role in regulating TCR-stimulated activation of the MAP kinase ERK in conventional T cells (35). In T-helper cells, AP-1 transcription factors have important effects on expression of the ICOS gene, which is downstream of both TCR/CD28 signaling and cytokine receptor signaling (36). Chang et al. claimed that TRAF3 regulates ICOS gene induction in Treg cells through activation of the ERK–AP-1 signaling pathway (34). We propose that TRAF3 mediates the differentiation of nTreg cells into TFR cells by regulating the expression of ICOS in nTreg cells.

As mentioned earlier, TFR cells are derived from Treg precursor cells. Sebastian et al. found that TFR cells were significantly reduced after knocking out the Helios gene in Treg cells in mice and that Helios regulates part of the suppressive function of Treg cells. They demonstrated that Helios molecules are involved in the differentiation of Treg cells into TFR cells (37). More research is needed to determine whether Helios is involved in the function of TFR cells. In a study of the role of mTOR in the differentiation of TFH, Yang et al. found that the TFR cells within the mouse spleen and lymph nodes were significantly reduced in mTOR knockout mice, suggesting that mTOR might be an important factor influencing the differentiation of TFR cells. However, the specific mechanism requires further study (38). In addition, there is no consensus regarding the procedure of emergence of circulating TFR cells. The overly simple explanation for the emergence of TFR cells in the circulation is that some TFR cells depart from GCs and then enter the blood. The complete differentiation of follicular T cells is generally considered to require a two-step process in which the initial activation of T cells occurs, mediated by DCs, followed by their interaction with B cells in the T-B border. An alternative proposed mechanism is that circulating TFR cells are generated from the initial steps that lead to the GC reaction in secondary lymphoid tissues and then exit the tissue before interacting with B cells. Circulating TFR cells can live *in vivo* for long periods, as can memory cells, and circulating memory-like TFR cells occupy less suppressive capacity than do dLN TFR cells. Moreover, circulating CXCR5+Foxp3+ T cells are ICOS−PD−1−Bcl−6−CD57−, which is different from tonsil TFR cells, and blood TFR cells have a distinctive naïve-like phenotype (18). It is likely that circulating TFR cells represent a pool of cells that are ready to be recruited into the B cell follicle after antigen re-exposure *via* migration toward CXCL13 (20). Many unknown factors might affect the differentiation and development of TFR cells, a possibility that warrants further study.

## TFR Cells Interact With B Cells and TFH Cells

**Figure 2:**
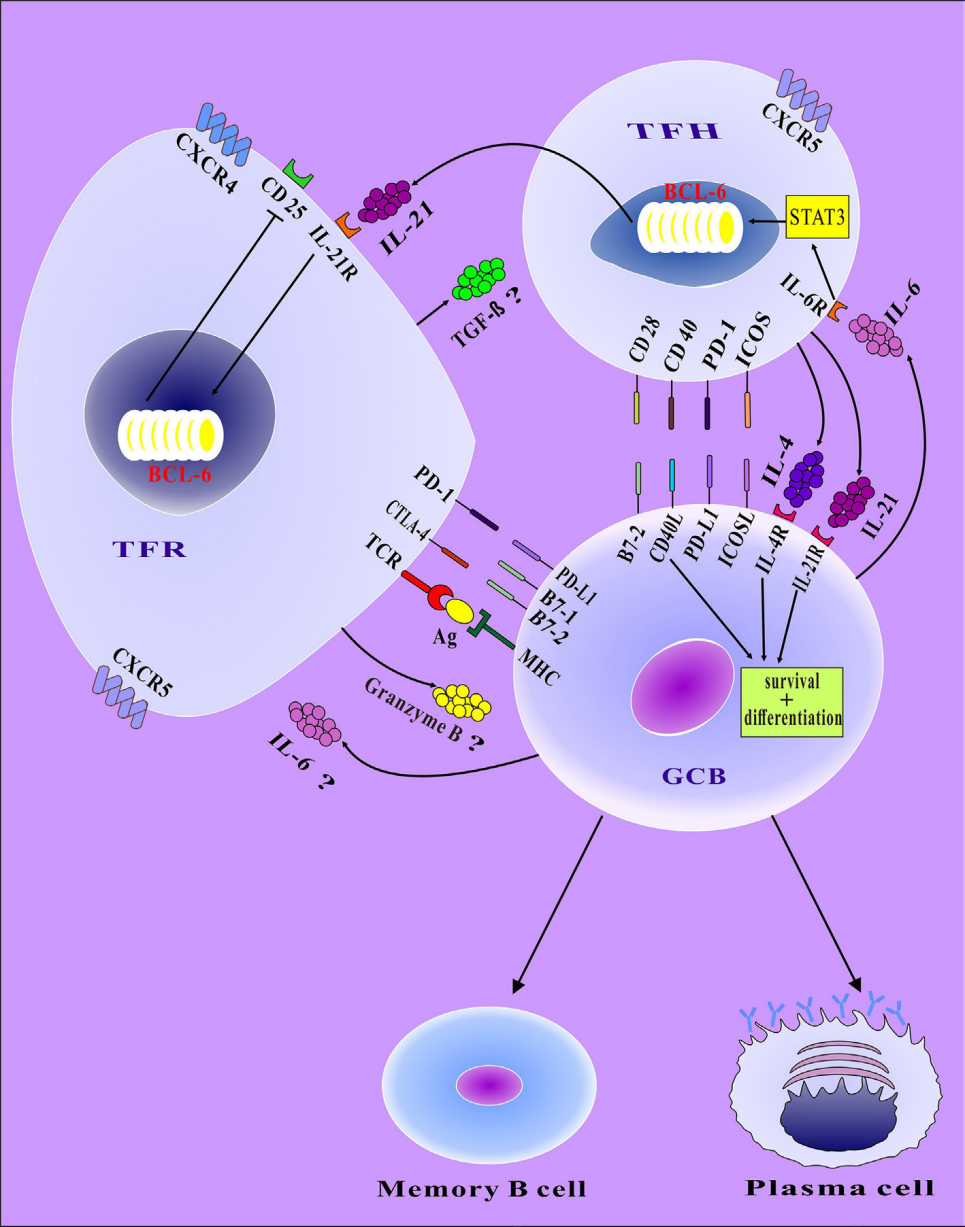
The mechanisms by which TFR cells interact with B cells and TFH cells. TFR cells can secrete granzyme B to induce B cell death or TGFβ to inhibit TFH cells. Moreover, changes in the shape of TFR cells allow TFR cells to mechanically interfere with the mutual contact between TFH and B cells. TFH cells provide survival and differentiation signals to B cells *via* the binding of CD40–CD40L and the secretion of both interleukin (IL)-4 and IL-21. TFH cells secrete IL-21 to reduce the expression of CD25 by upregulating the BCL-6 of TFR cells. Cytokines produced by B cells (IL-6) rely on STAT3 to regulate the expression of BCL-6 and thereby promote both the differentiation of TFH cells and the secretion of IL-21 by TFH cells. In addition, the IL-6 secreted by B cells may act on TFR cells, the effects of which are unclear. Cell-cell interactions between TFH cells and B cells (e.g., involving CD28–B7-1, ICOS–ICOSL, PD-1–PD-L1, CD40–CD40L) play fundamental roles in TFH cell functions. In the germinal center, GCB cells differentiate into memory B cells and plasma cells; *via* interaction with TFH cells, these cells are stimulated to undergo activation, class switch recombination, and affinity maturation.

There is consensus that TFR cells can alter their function through their interaction with B cells and TFH cells (Figure 2). In a study of the effects of TFR cells on TFH cells, SRBC was used to immunize mice that had the BCL-6 gene knocked out in FOXP3+ T cells. The TFR cells in the spleens of mice were found to be significantly downregulated after immunization, resulting in the significant upregulation of IL-10, IL-21, IFN-γ, and other cytokines secreted by TFH cells. In addition, IgG antibody in serum decreased, and IgA antibody increased. It was confirmed that TFR cells were capable of affecting the expression of certain cytokines in TFH cells. In this way, the interaction between TFH cells and B cells could be affected, and the corresponding antibody could be changed (39). Moreover, after knockout of CTLA-4 in TFR cells, the number of TFR cells increased both *in vivo* and vitro, but their inhibitory function was impaired, whereas TFH cells, B cells, and the antibody increased. These findings indicated that CTLA-4 plays a critical role in the interactions among TFR, TFH, and B cells (32).

Other mechanisms might be involved in the regulation of TFR cells to TFH cells and B cells. For example, TFR cells may regulate the GC reaction by secreting IL-10, TGF-β and other cytokines. In general, IL-10 is considered an inhibitory cytokine; however, it has been resported that TFH cells, like TFR cells, can produce IL-10 (40). The potential of IL-10 secretion as a way for TFR cells to regulate the GC response requires further study. In addition, a study has found that TFR cells suppress the production of antibody by limiting the magnitude of the GC reaction (14). However, Laidlaw et al. found that TFR cells can also promote the GC reaction *via* the secretion of IL-10, which promotes the expression and activity of FOXO1 in activated B cells. This promotion facilitates the adoption of a dark zone (DZ) phenotype by GC B cells and potentially enhances affinity maturation after acute infection with LCMV (41). The GC consists of two compartments that exhibit distinct functions and topographies: the DZ, where proliferative expansion and Ig somatic hypermutation occur; and the light zone, where high-affinity antibodies are produced by B cells that were selected in response to signals supplied by follicular dendritic cells and TFH cells and where B cells undergo class switch recombination (42–44). These studies suggest that TFR cells play a many-sided role in the fine-tuning of the GC reaction. However, many questions remain in the following areas: 1. Some researchers have confirmed that TFR cells can promote the production of antibodies in the presence of exogenous antigens in mice. However, it is unclear whether TFR cells exhibit analogous phenomena in the presence of endogenous antigens. 2. TFR cells can promote or inhibit the production of antibodies; however, the mechanism that underlies the promotion vs. inhibition of TFR cells is unknown. (Potentially, the character of the antigen is involved). 3. During the promotion of antibody production by TFR cells, the effect of these cells on TFH cells (e.g., inhibition, promotion, or neither) is unclear. It is possible that TFR cells play a role in the GC reaction as a “seesaw balancer.” We hypothesize that TFR cells play different roles under different conditions in the body. Furthermore, given the requirement of the innate character of immunity, TFR cells have inhibitory effects when the GC reaction is strong relative to the needs of the organism but play a facilitating role when the GC reaction is weak relative to the needs of the organism. TFR cells play a role in inhibiting or promoting the GC reaction through different mechanisms and ultimately do so to maintain immune homeostasis. TGF-β is likely to be the cytokine *via* which TFR cells regulate the GC reaction. It has been reported that TGF-β can inhibit TFH cells and B cells to regulate the GC reaction (45). TFR cells secrete granzyme B to induce cell death. Zhao et al. confirmed that Treg cells can induce B cell death by secreting granzyme B (46). It has also been found that TFR cells are similar to Treg cells, which can express a certain amount of granzyme B (16).

TFR cells can inhibit glucose metabolism in B cells by inhibiting their Myc and mTOR signaling pathways. TFR cells do not only affect the glucose metabolism of B cells but also affect intracellular one-carbon metabolism, and serine biosynthetic pathway-related genes have been found to be significantly reduced in repressed B cells compared to activated B cells. These metabolic changes eventually lead to B cell epigenetic changes that persist even when B cells are distant from TFR cells (47).

TFR cells may inactivate AKT in B cells. The aberrant activation of the Akt signaling pathway affects the development, activation and proliferation of B cells (48, 49). It was found that normal TFR cells that were transferred into mice were capable of inactivating AKT by downregulating the phosphorylation of AKT in the B cells of the spleen to a higher level than were TFR cells whose capacity of suppression was damaged (30).

TFR cells mechanically interfere with the mutual contact between TFH and B cells. A strong TCR signal can lead to a “stop” signal that can limit the movement of T cells such that T cells are exposed to antigen-presenting cells for a long period of time (50). It has been speculated that TFR cells that lack PD-1 can produce a strong TCR signal, which elicits a “stop” signal. The “stop” signal restricts T-cell movement, prompting TFR cells to prolong the time of interaction with B cells (13, 51).

TFR cells suppress the IL-1-induced activation of TFH cells. IL-1 treatment can promote the proliferation of TFH cells *in vivo* and activate TFH-cell production of IL-4 and IL-21 by interacting with the IL-1R1 agonist receptor of TFH cells *in vitro*. CD4+CXCR5+PD-1+Bcl6+Foxp3+CD25− TFR cells that express the IL-1 decoy receptor IL-1R2 and the IL-1 receptor antagonist IL-1Ra were found to suppress the IL-1-induced activation of TFH cells as efficiently as could the IL-1 receptor antagonist Anakinra (52). The regulatory mechanism of TFR cells acting on TFH cells and B cells might involve more processes than are mentioned in this review and requires further study.

## TFR Cell Regulation

Many cell-surface molecules and signal pathways are required for TFR development. Substances that can interact with the surface and intracellular signal pathway molecules of TFR cells during the differentiation and development of TFR cells have significant effects on the development and function of TFR cells.

An increasing number of researchers studying TFR cells believe that altering the relevant cytokines or signaling pathways involved in the differentiation and development of TFR cells can affect the number, proportion or function of TFR cells and thereby affect the GC reaction. A decrease in the expression of Id2 and Id3 during TCR-mediated TFR cell signaling was found to lead to an increase in binding activity to E protein (which can bind to the corresponding DNA sequence as a transcriptional activator or inhibitor), thereby inducing the expression of specific genes of TFR cells, such as CXCR5 and IL-10. An increase in Id2 and/or Id3 abundance, which is required for the differentiation of TFR cells, likely indirectly modulates Bcl-6 and Blimp-1 protein abundance and ultimately leads to the development of a mature TFR cell population (53).

Several cytokines have strong effects on T cells in their differentiation microenvironment. TGF-β possesses the capacity of inducing the differentiation of conventional CD4+ T cells into Treg cells and the development of Treg cells in the thymus.

Interleukin-2 promotes the phosphorylation of STAT5 and thus maintains the expression of FOXP3 molecules on Treg cells and the proliferation of Treg cells. TGF-β and IL-2 also have strong effects on TFR cells. It was reported that TGF-β and IL-2 promote the differentiation of Treg precursor cells into TFR cells (54). However, the effects of IL-2 on TFR cells are complex. It was found that high IL-2 concentrations at the peak of infection prevented TFR cell development by a Blimp-1-dependent mechanism. Furthermore, when the virus was dispelled and IL-2 concentrations decreased, some CD25hi Treg cells downregulated CD25, upregulated Bcl-6 and differentiated into TFR cells, which migrated into the B cell follicles to avoid the accumulation of self-reactive B cell clones (55). The results of the above two studies are inconsistent, and the reason for this discrepancy might be related to the concentration of IL-2. In the experiment of Botta et al., Foxp3+ T cells that were cultured at low IL-2 concentration were able to upregulate the expression of Bcl-6 and CXCR5 as well as increase the number of TFR cells (55). We hypothesize that a low concentration of IL-2 is necessary for the differentiation of TFR cells and that the differentiation of TFR cells will become impaired as the concentration of IL-2 increases in the microenvironment of cell differentiation.

The IL-21 that is secreted by TFH cells interacts with B cells, thereby promoting the proliferation of B cells, the differentiation of plasma cells and the production of antibodies (56). However, it has an inhibitory effect on TFR cells (57, 58). IL-21 may also reduce the expression of CD25 by upregulating BCL-6 in TFR cells and thus attenuating the affinity of TFR cells for IL-2. This process would then limit the proliferation of TFR cells and lead to changes in the number of TFR cells and ultimately an enhanced GC response (57, 58) (Figure 2). Sage et al. demonstrated that TFR cells are capable of selectively and continuously inhibiting B cell metabolism and antibody production. However, IL-21 can reverse the inhibitory effect of TFR cells on B cells (47).

Store-operated Ca^2+^ entry (SOCE) of extracellular fluid through the Ca^2+^ release-activated Ca^2+^ (CRAC) channel for entering the intracellular fluid constitutes the major source of Ca^2+^ signaling in T lymphocytes. CRAC channels are activated after receiving the TCR signal, resulting in inositol-1,4,5-triphosphate (IP3) production and the release of Ca^2+^ from the endoplasmic reticulum (ER) Ca^2+^ stores. ER proteins stromal interaction molecule 1 (STIM1) and STIM2, which are combined with ORAI1 on the plasma membrane, allow the monitoring of the concentration of intracellular Ca^2+^. ORAI1 is a pore-forming subunit of the CRAC channel, which is bound to STIM1 and STIM2 to allow STIM1 and STIM2 to mediate sustained SOCE in T cells (59). Vaeth et al. reported that SOCE regulation of the differentiation of TFH and TFR cells relies on SOCE regulation of the expression of transcription factors, including IRF4, BATF and BCL-6, by NFAT (60). When mice were infected with LCMV, the absence of SOCE inhibited the differentiation of TFH cells and TFR cells, resulting in an impaired GC response and eventually an inadequate humoral immune response. In mice under noninfectious conditions, the deletion of SOCE resulted in a significant decrease in TFR cells, which resulted in a spontaneous GC reaction and ultimately led to an autoimmune reaction accompanied by the production of large amounts of autoantibodies (60).

ICOS in T cells induces PI3K ligand p85α to interact with OPN-i such that OPN-i moves into the nucleus to prevent the degradation of BCL-6 in an ubiquitination-dependent manner (61). The previously discussed BCL-6 plays an important role in the differentiation of TFR cells. TFR cells were greatly reduced in mice when the OPN-i gene was knocked out. The p85-OPN-i pathway has been confirmed to play a bridging role in the process by which ICOS activates BCL-6 to induce TFR differentiation (61). IL-6 relies on STAT3 to regulate the expression of BCL-6 in TFH cells, thus promoting the differentiation of TFH cells and the secretion of IL-21 by TFH cells (62–65). Moreover, the aberrant expression of IL-6 is an important factor in the development of many autoimmune diseases (66). It has been documented that IL-6 possesses the capacity to downregulate FOXP3 transcription factors and proteins in nTreg cells, which results in the loss of inhibitory function of Treg cells (67). IL-6 inhibits the differentiation of immature T cells into Treg cells (28). Therefore, we speculate that in the process of the development of autoimmune diseases, IL-6 likely has a direct or indirect effect on TFR cells and thereby promotes the occurrence of disease. The differentiation and function of TFR cells are affected by many factors. Changes in the relevant factors change will have strong influences on TFR cells. A deeper understanding of TFR cells has the potential to provide insight into new ways to intervene in TFR cells for the treatment of related diseases.

## Involvement of TFR Cells in Autoimmune Diseases

TFH cells interact with B cells by secreting cytokines such as IL-21 and IL-6 or by providing a variety of activation signals for B cells, which promote GCB-cell production of a large number of autoantibodies. TFH cells thus participate in the occurrence and development of autoimmune diseases (68). Dysfunction of TFR cells in the GC reaction affect the large amounts of high-affinity autoantibodies produced by B cells and thus plays a role in the occurrence of a variety of autoimmune diseases (69–71).

It has been reported that the immune homeostasis of TFR cells and TFH cells is disrupted in the peripheral blood of patients with autoimmune diseases (Table 1). In peripheral blood from myasthenia gravis (MG) patients, the frequency of TFR cells was found to be downregulated, whereas that of TFH cells was found to be upregulated. The frequency of TFR cells was negatively correlated with the clinical pathological score. After receiving effective treatment, the patients’ peripheral blood TFH cells and TFR cells gradually restored the immune homeostasis. Moreover, the clinical pathological score of patients also declined, and the patients’ condition improved gradually (72). However, the researchers of that study did not explore the specific causes of this phenomenon in detail.

**Table 1:** Involvement of TFR in blood from patients with autoimmune diseases.

Disease	Phenotype of TFR cells	Dysfunction of TFR cells	Reference
Sjögren syndrome	CD4+CD25+FOXP3+CXCR5+	TFR↑, TFH↓, TFR/TFH↑	(18)
Rheumatoid arthritis	CD4+CD25+CD127-CXCR5+	No change	(40)
Myasthenia gravis	CD4+CXCR5+FOXP3+	TFR↓, TFH↑, TFR/TFH↓	(72)
Multiple sclerosis (MS)	CD4+CD25+CD127-CXCR5+PD-1+	TFR↓, TFH↑, TFR/TFH↓	(73)
MS	CD4+CXCR5+FOXP3+	No change	(74)
Systemic lupus erythematosus	CD4+CD25+CD127low-intCXCR5+	TFR↓, TFH↑	(75)
Child immune thrombocytopenia	CD4+FOXP3+CXCR5+ICOS+	TFR↓, TFH↑	(76)
Henoch–Schönlein purpura in children	CD4+Foxp3+CXCR5+ICOS+	TFR↓, TFH↑	(81)
Ankylosing spondylitis	CD4+Foxp3+CXCR5+	TFR↑, TFH↑, TFR/TFH↑	(82)

A similar phenomenon was observed in multiple sclerosis (MS) patients. The frequency of TFR cells was reported to be downregulated in the peripheral blood of patients with MS, whereas the frequency of TFH cells was upregulated, and the rate of TFR/TFH was decreased. Moreover, the inhibitory function of TFR cells was decreased significantly, and this decrease in inhibitory capacity was due to a marked increase in TH17-like TFR cells. The decrease in the frequency of TFR cells in the peripheral blood of MS patients might be due to the return of TFR cells of the peripheral circulation to the lymphatic organs to inhibit the enhanced GC reaction (73). It has been reported that memory TFR cells in the peripheral circulation are activated by reantigen exposure and can be returned to the secondary lymphoid organs to enter the GC and reregulate the GC reaction and can enter peripheral tissues such as skin (20). In addition, Jones et al. found that the frequencies of TFR, TFH and Treg cells in the peripheral blood of patients with MG during the phase known as clinically isolated syndrome (CIS) showed no significant differences from those of healthy controls. However, TFR and Treg cells can be divided into three functionally distinct “fractions” based on CD45RA and FoxP3 expression. In one study, these fractions (Fr) were depicted as resting TFR and Treg cells (FrI: CD45RA+ FoxP3lo), activated TFR and Treg cells (FrII: CD45RA− FoxP3hi) and cytokine-producing non-TFR and non-Treg cells (FrIII: CD45RA− FoxP3lo). The authors of that study found lower percentages of FrI Treg and TFR cells and higher percentages of FrIII non-Treg and non-TFR cells in the CIS group than in the control group. Moreover, levels of Helios protein expression of all Treg and TFR fractions were significantly lower in the CIS group. These findings suggest that CIS is associated with a pool of regulatory cells that lack suppression capacity and might have a proinflammatory function (74). The reason for the inconsistent results of the two studies of the TFR cells in the peripheral blood of MS patients might be due to the use of different definitions to label TFR cells or the analysis of specimens from MS patients at different periods of pathogenesis.

Xu et al. showed that in the peripheral blood of patients with systemic lupus erythematosus (SLE), the frequency of TFR cells was downregulated and the frequency of TFH cells and the level of plasma IL-21 were upregulated. In addition, the severity of the disease, the level of plasma IL-21, and the level of serum anti-dsDNA antibody were negatively correlated with both the frequency of TFR cells and the TFH/TFR ratio. After treatment, the frequency of TFR cells was higher than before, the frequency of TFH cells was lower than before, the severity of the disease and the level of anti-ds-DNA antibody decreased, and the patient’s condition improved (75). Wu et al. found that the level of serum anti-ds-DNA IgA antibody was higher in autoimmune lupus model mice, which lack TFR cells, than in WT lupus model mice. This result demonstrated that TFR cells can inhibit the production of some autoantibodies related to autoimmune diseases. In addition, imbalance of the TFH/TFR ratio was found to be significantly correlated with the occurrence of SLE (39).

In the peripheral blood of children with immune thrombocytopenia (ITP), the frequency of TFR cells in the peripheral blood decreased, the frequency of TFH cells increased, the level of plasma IL-2 decreased, and the level of plasma IL-21 increased. It was discovered that in the peripheral blood of patients with ITP, the expression of Bcl-6 and C-MAF mRNA in CD4+ T cells was upregulated, the level of Blimp-1 mRNA in CD4+ T cells was downregulated, and PD-1 mRNA levels were upregulated (76). Bc1-6 and c-MAF can increase the expression of CXCR5, PD-1, ICOS and other cell molecules in CD4 T cells, which induce the differentiation of T cells into TFH cells (22, 77, 78). Therefore, these related changes might be associated with the increase in the frequency of TFH cells in the peripheral blood of ITP patients. Because PD-1 can inhibit the development of TFR cells, the increased levels of PD-1 mRNA in CD4+CD25+ T cells might be responsible for the decrease in TFR cells. Alternatively, the decrease in TFR cells might be due to a decrease in the level of IL-2 and an increase in the level of IL-21. This contributes to the decrease of TFR cell frequency in the peripheral blood of patients with ITP and the increase of TFH cell frequency in peripheral blood of patients with ITP. Because IL-2 can promote the development of TFR cells and inhibit the development of TFH cells, IL-21 can promote the development of TFH cells and inhibit the proliferation of TFR cells (54, 57, 79, 80). After effective treatment, the condition of patients with IPT was improved, the frequency of TFR cells was increased, and the frequency of TFH cells was decreased.

Not all types of autoimmune diseases show similar phenomena, i.e., an increase in the frequency of TFR cells and a decrease in the frequency of TFH cells in the peripheral blood. In the peripheral blood of ankylosing spondylitis (AS) patients, the frequency of TFR cells and TFH cells were both upregulated, and the TFR/TFH ratio was increased. Moreover, the level of serum IL-21 was increased, and the level of serum IgA showed a negative correlation with the frequency of TFR cells. After 1 month of standardized treatment, the frequency of TFR cells in peripheral blood of the patients was significantly higher than it was before treatment, and the level of serum IL-21 was significantly lower than before. In addition, the ratio of TFR/TFH was increased, and the level of serum IL-21 was negatively correlated with the frequency of TFR cells. However, the frequency of TFH cells did not significantly differ between before and after treatment (81). A possible explanation for the increased frequency of TFR cells in these patients is that the increased frequency of TFH cells might have led to compensative spontaneous regulation of the immune system to maintain the immune homeostasis of the GC reaction. An alternative interpretation is that the change in the microenvironment of the development of Treg precursor cells caused the disease. In this scenario, TFR cells are unable to enter GCs to differentiate into a complete form such that a large number of immature TFR cells gather in the peripheral blood, which causes the frequency of TFR cells to increase in the peripheral blood of patients with AS.

In the peripheral blood of Sjögren syndrome (SS) patients, the frequency of CD4+ T cells was not significantly different from the frequency in healthy controls, and the absolute number of CD4+ T cells was decreased. Furthermore, the frequency of TFH cells was decreased and the absolute number of TFH cells showed no significant difference. The frequency of TFR cells was increased significantly, whereas the absolute number of TFR cells was decreased. Moreover, the TFR/TFH ratio was increased, affecting the production of SS-related autoantibodies (18).

TFR and TFH cells are not necessarily involved in all autoimmune diseases or in each pathological stage of a disease. It was reported that the frequencies of TFH cells and TFR cells were not significantly different from those in peripheral blood in the early stage of RA in patients in the United States (40, 56). However, marked differences have been observed in different animal models. Kim et al. used BXD2 mice, which produce large amounts of rheumatoid factor and autoantibodies and develop aggressive arthritis on their own. Compared with the spleen of WT mice, the spleen of BXD2 mice showed an increased frequency of TFH cells. Although the number of TFR cells were increased in BXD2 mice, the frequency of CD4+FOXP3+T cells was decreased in these mice, and the GC B/TFR and TFH/TFR ratios were upregulated. In addition, the level of serum IL-21 was also significantly increased in BXD2 mice. These observed changes in the TFR cells of BXD2 mice and their associated pathologic symptoms suggest that TFR cells are likely involved in the pathogenesis of RA (82).

The pathological features of several autoimmune diseases suggest that TFR cells are likely related to the occurrence and development of these diseases. Although the occurrence of disease is not necessarily due to the downregulation of TFR cells, it is likely that normal immune homeostasis is impaired due to abnormalities in TFR cells and TFH cells; this impairment leads to the abnormal levels of cytokines associated with autoimmune diseases and an increase in autoantibody production. This production ultimately exceeds the body’s immune tolerance limit and leads to the emergence of autoimmune disease.

There is a hypothesis that if the immune balance between TFR cells and TFH cells is restored in an autoimmune disease, it can reduce or reverse the development of the autoimmune disease. Experimental autoimmune myasthenia gravis (EAMG) is an animal model for human MG, which is induced in Lewis rats by immunization with acetylcholine receptors (AChR) in complete Freund’s adjuvant and developed into a B cell-mediated, T cell-dependent, complements-involved autoimmune disease (83). It was found that the frequency of TFH cells increased and that of TFR cells decreased during the onset of EAMG. After intraperitoneal injection of ATRA into the EAMG rats, the serum anti-AChR antibody IgG decreased, the frequency and absolute number of TFH cells in the rats decreased, the frequency and number of TFR cells increased, and the clinical symptoms of the EAMG model rats were alleviated (84). In another study, caspase-1 inhibitors were injected into EAMG model mice. Compared with EAMG model mice without injection, injected mice showed a decreased frequency of TFH cells and an increased frequency of TFR cells. In addition, in the injected EAMG mice (85), GC B cells were decreased, the GC reaction was decreased, and the numbers of self-reactive antibodies were reduced in the lymph nodes. These results suggest that the severity of an autoimmune disease can be alleviated by interfering with the number or frequency of TFR cells and TFH cells.

In summary, most of the current studies on TFR cells in autoimmune diseases investigate TFR cells only in peripheral blood. In addition, technical methods are lacking for exploring the TFR cells of GCs in patients with autoimmune diseases, and uniform biomarkers for detecting TFR cells are lacking. As a result, some studies might not address the performance of “complete TFR cells” in autoimmune diseases. The GC reaction plays an extremely important role in the humoral immune response. TFH cells help B cell development and the GC reaction through direct interaction and/or cytokine secretion, which leads to the maturation of B cells and the production of high-affinity antibodies to resist exogenous or endogenous antigens. TFR cells play a role in limiting the “help” provided by TFH cells, thus maintaining the balance of the GC reaction. However, dysfunction of TFR cells may lead to the loss of immune homeostasis and autoimmunity. Based on our current understanding of the interactions of TFR and TFH or B cells and the significance of TFH and B cells in the pathogenesis of autoimmune diseases, we speculate that TFR cells are likely involved in the pathogenesis of autoimmune diseases. As mentioned above, there are many regulatory factors, such as cytokines, ligands-receptors, and transcription factors, which can affect the number or function of TFR cells. Modulation of this relationship to reshape the proportion and function of TFR cells and TFH cells could help an organism recover its immune homeostasis and might represent a new option for the clinical treatment of autoimmune diseases.

## Author Contributions

YG wrote the manuscript and discussed the content with the other authors. JT discussed the content with the other authors. SW conceived the topic and revised the manuscript.

## Conflict of Interest Statement

The authors declare that the research was conducted in the absence of any commercial or financial relationships that could be construed as a potential conflict of interest.
